# Supplementary material for: Allergen-induced NLRP3/caspase1/IL-18 signaling initiate eosinophilic esophagitis and respective inhibitors protect disease pathogenesis
Source: Commun Biol. 2023 Jul 31;6:763. doi: 10.1038/s42003-023-05130-4 (PMC10390481; doi:10.1038/s42003-023-05130-4)
Supplement: Supplementary file 2 — Supplementary Information [file 42003_2023_5130_MOESM2_ESM.pdf]

Supplementary Figure 1

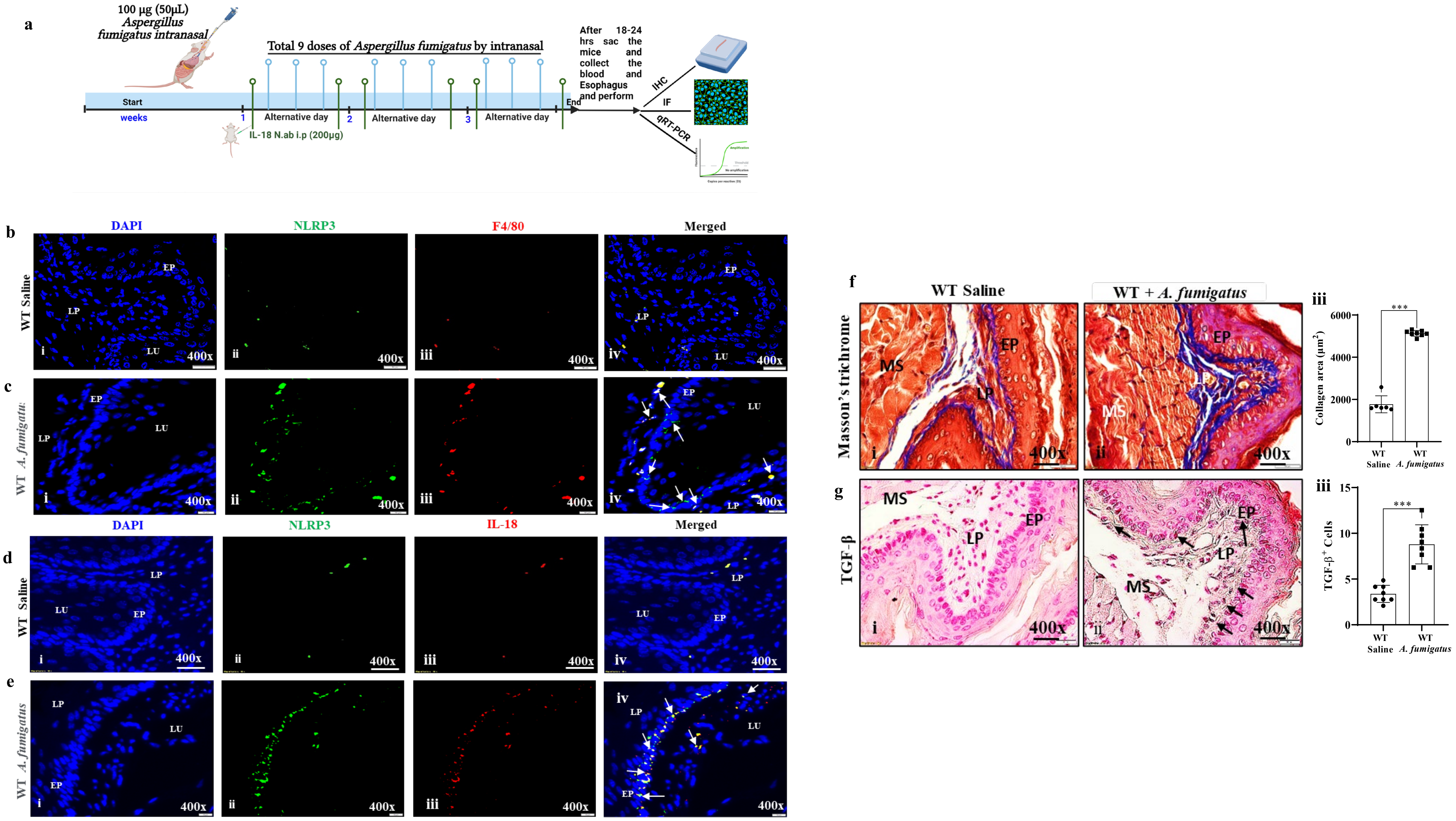

Supplementary Figure 2

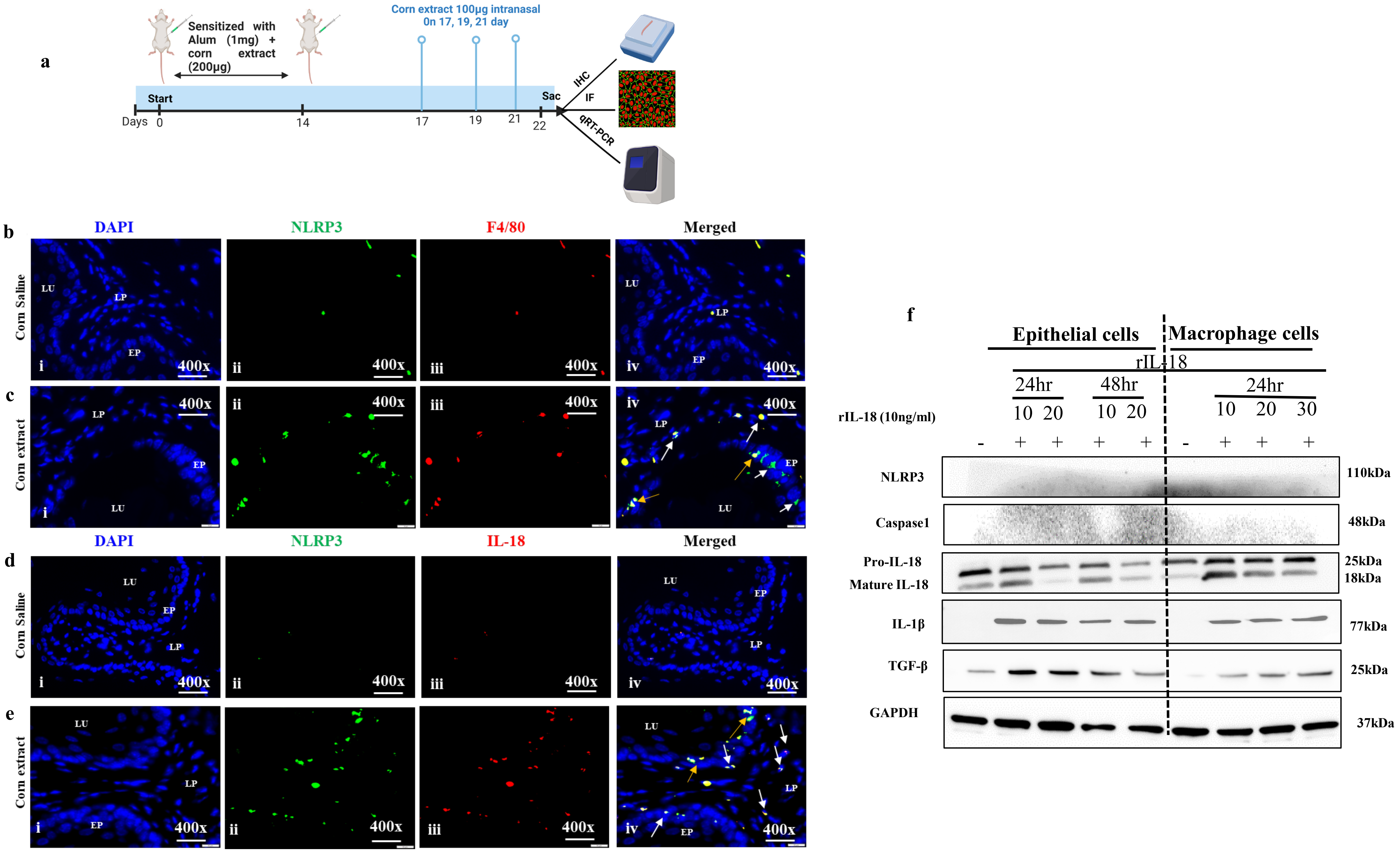

Supplementary Figure 3

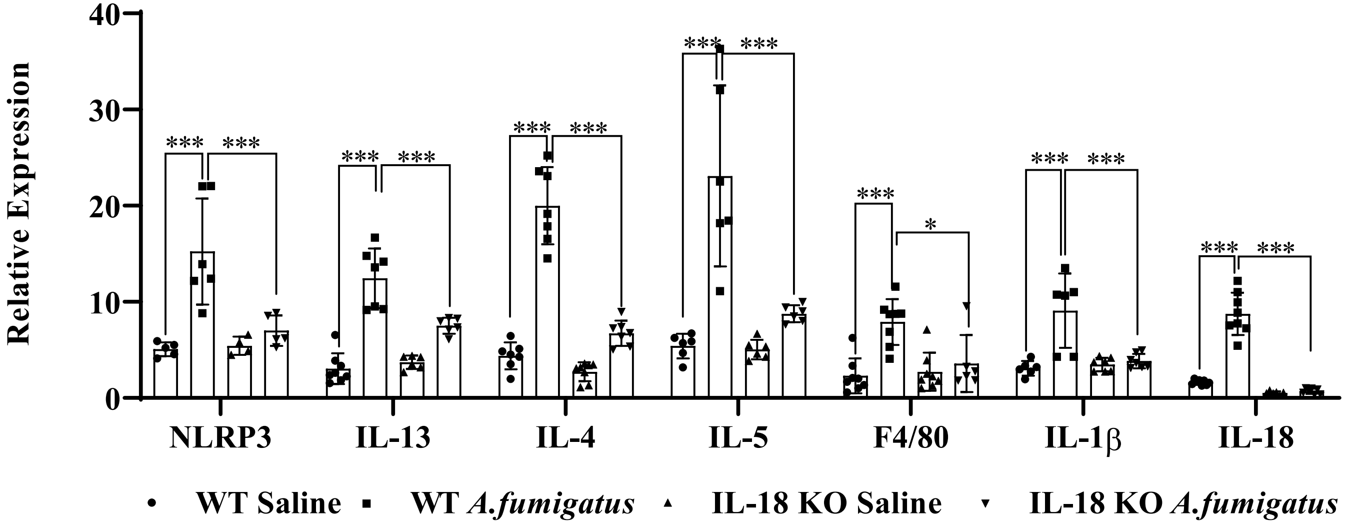

Supplementary Figure 4

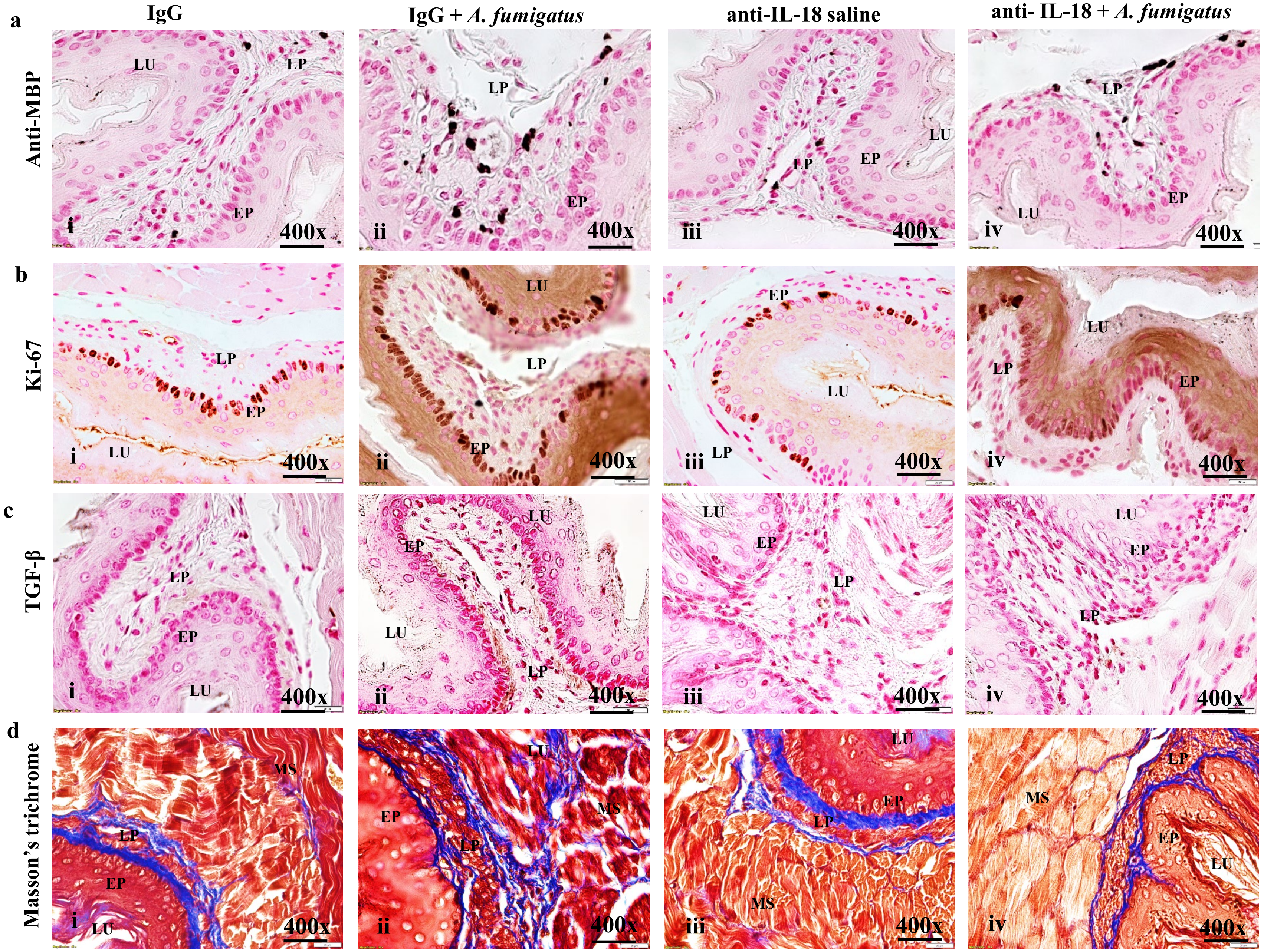

Supplementary Figure 5

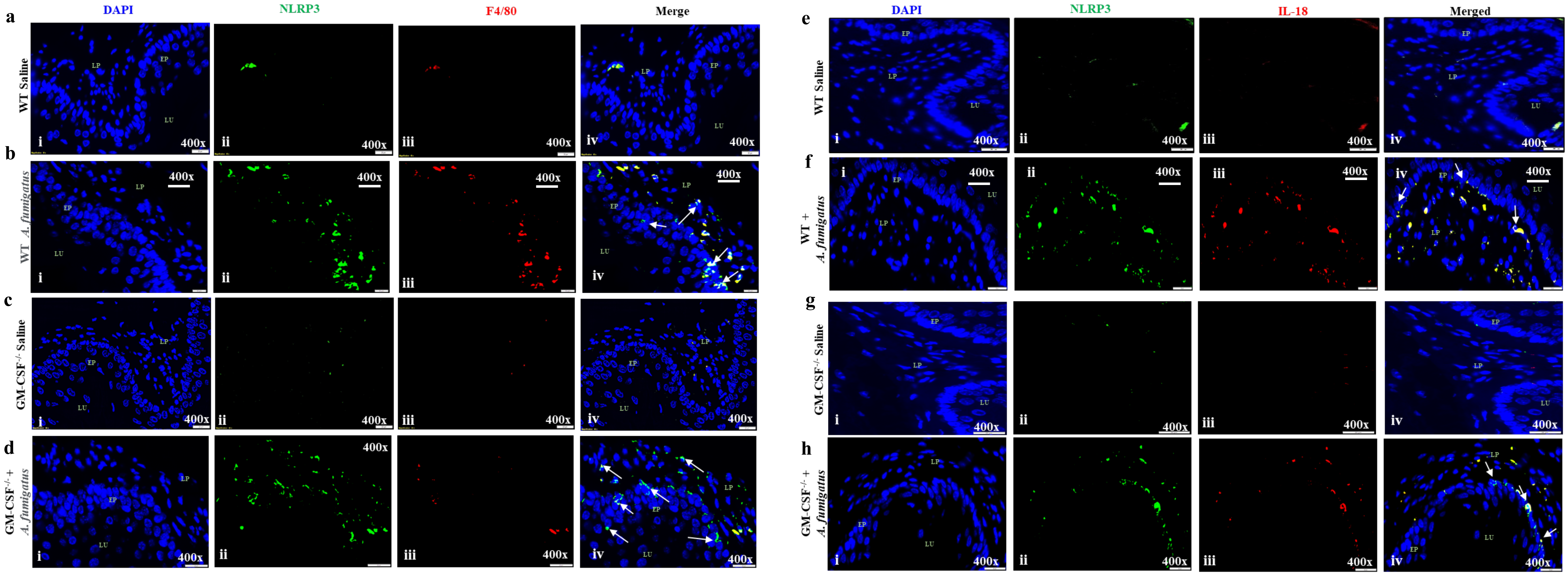

Supplementary Figure 6

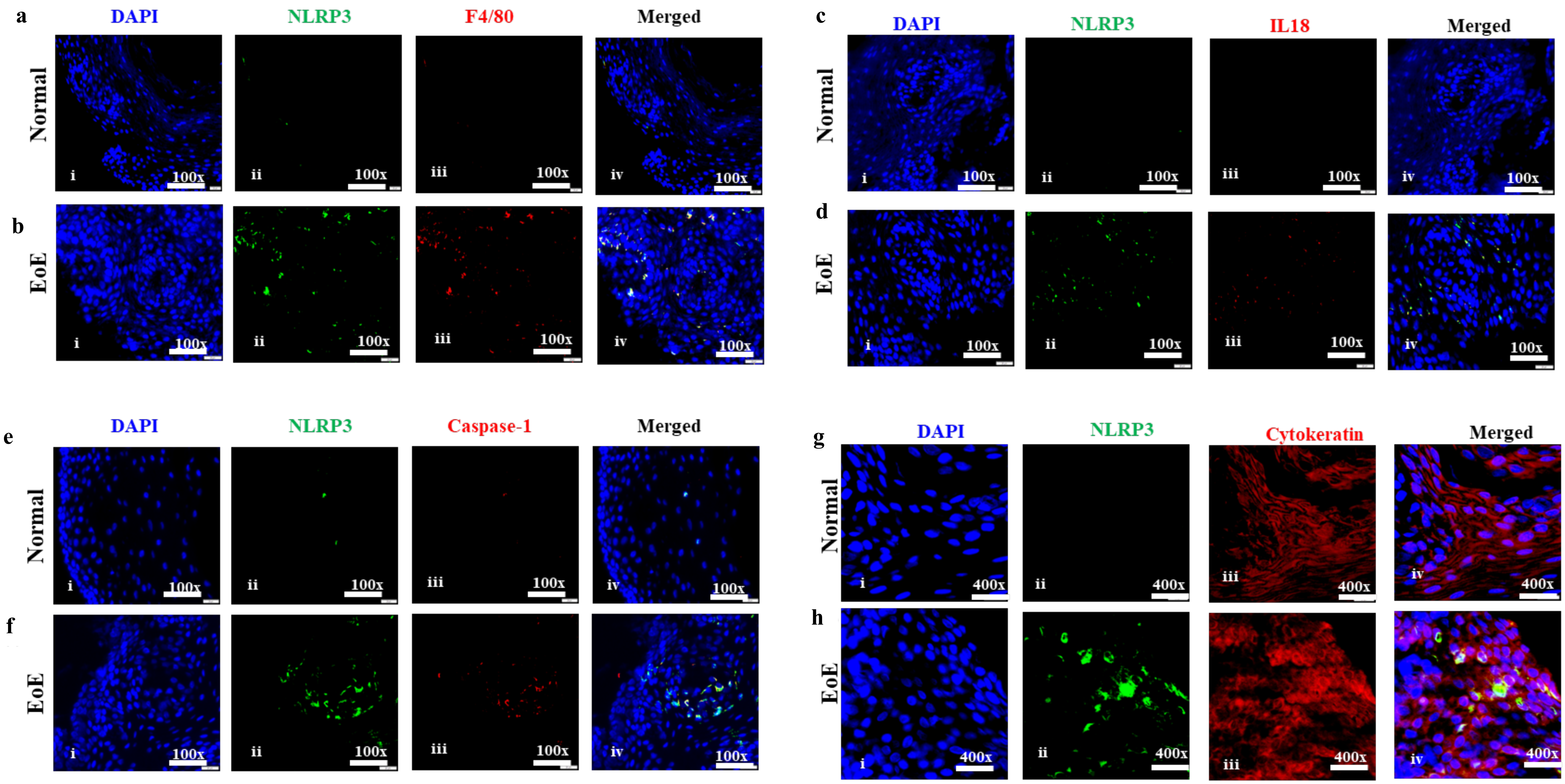

Supplementary Figure 7

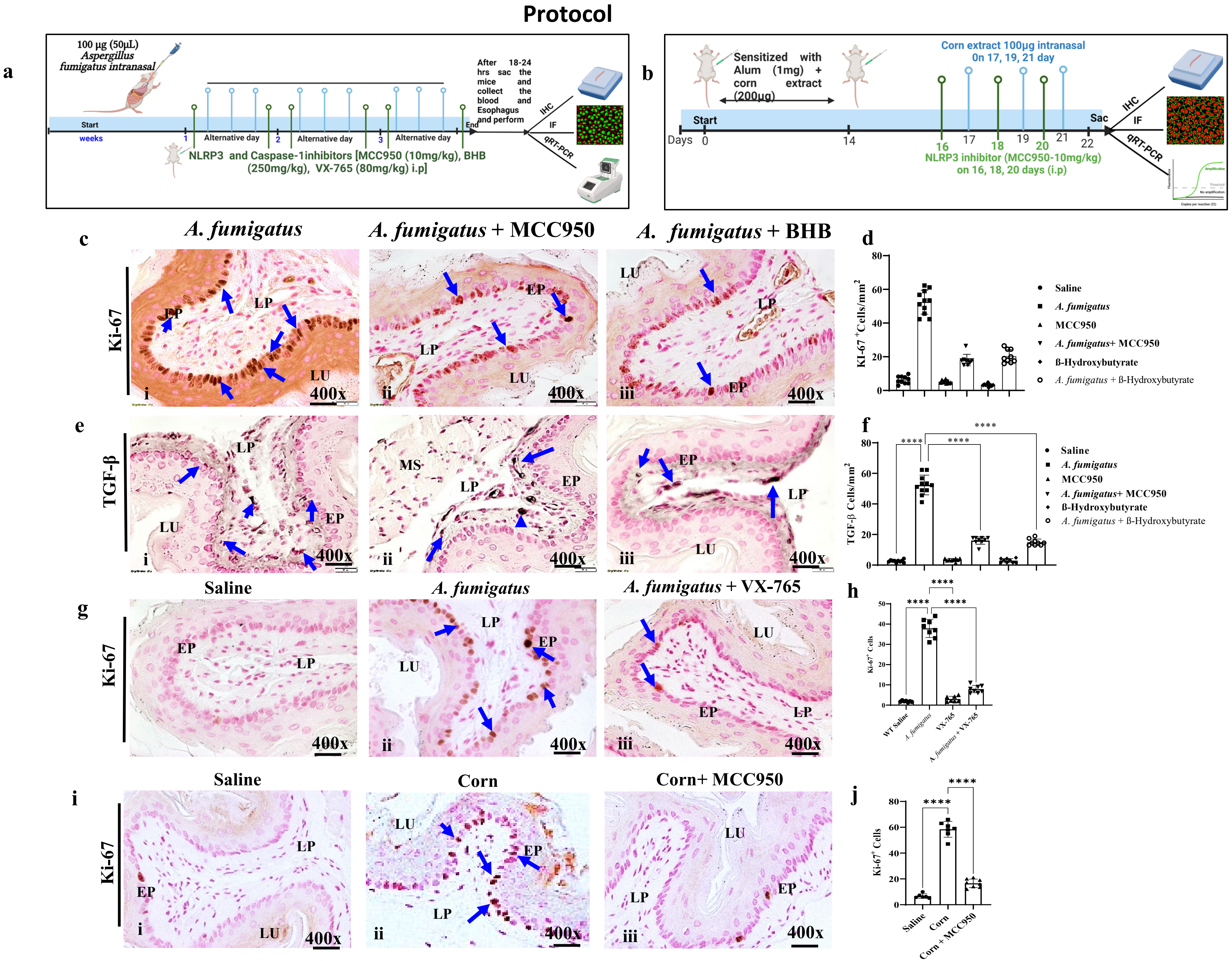

Supplementary Figure 8

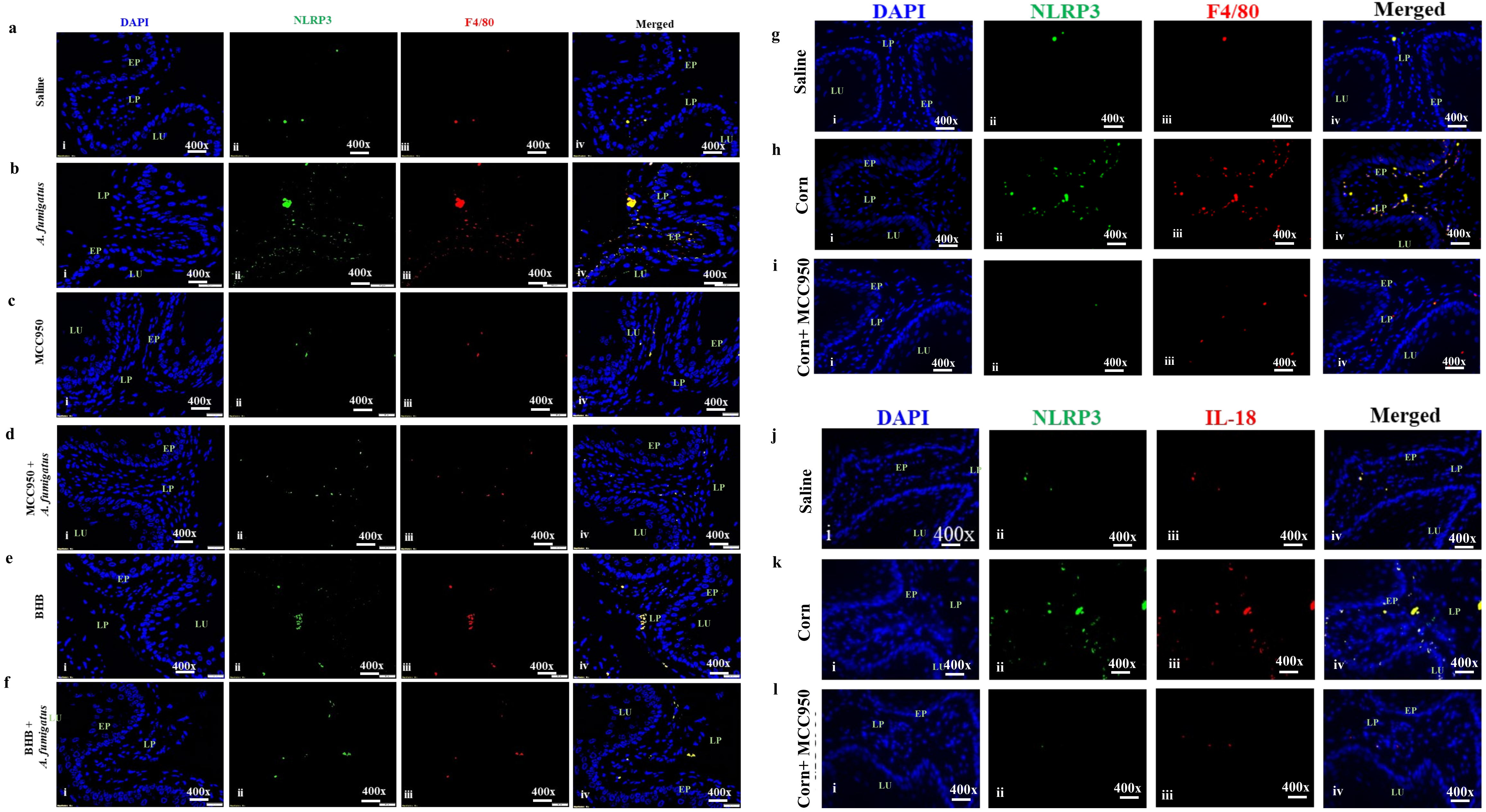

Supplementary Figure 9

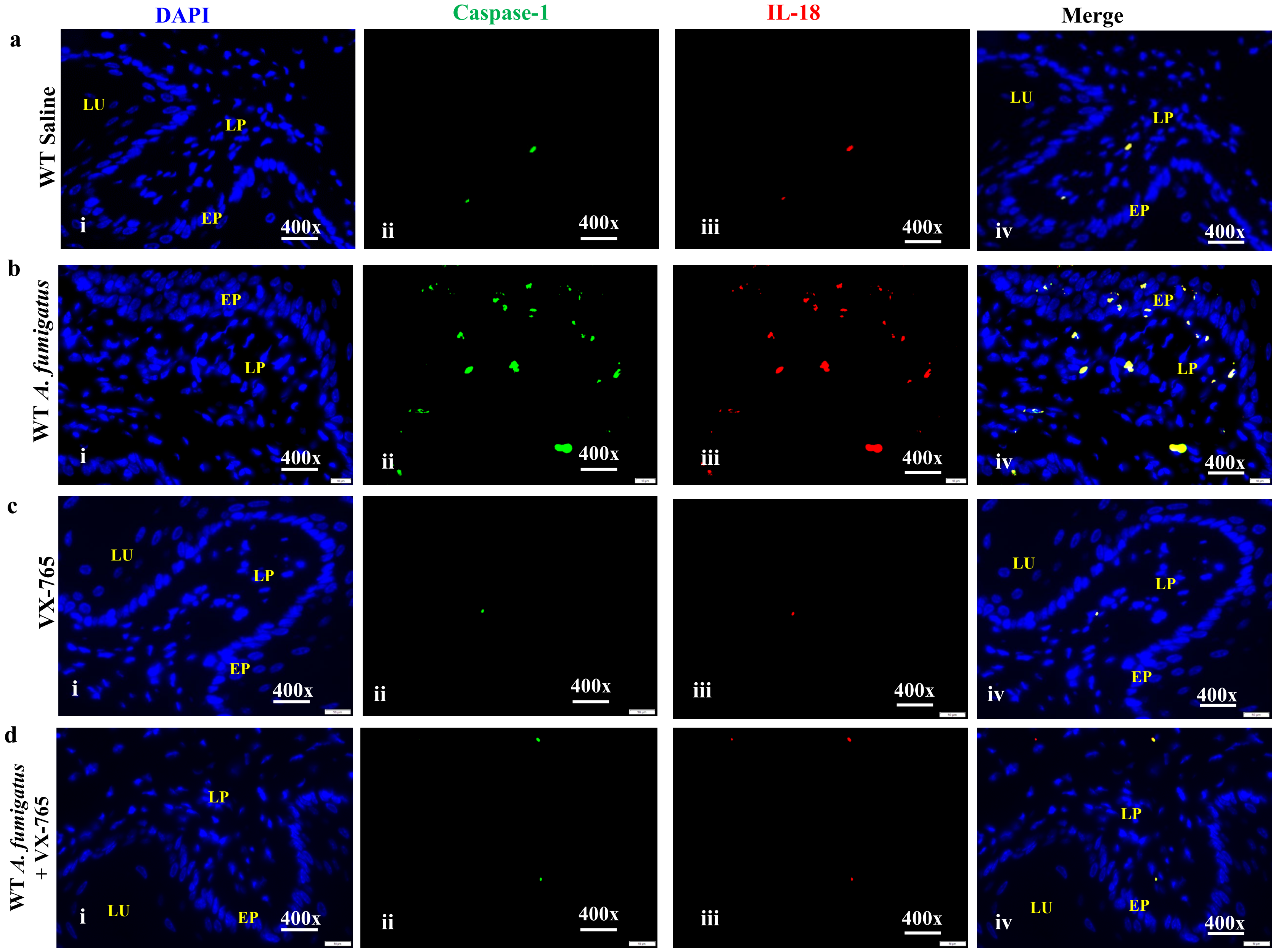

Supplementary Figure 10 Original blots

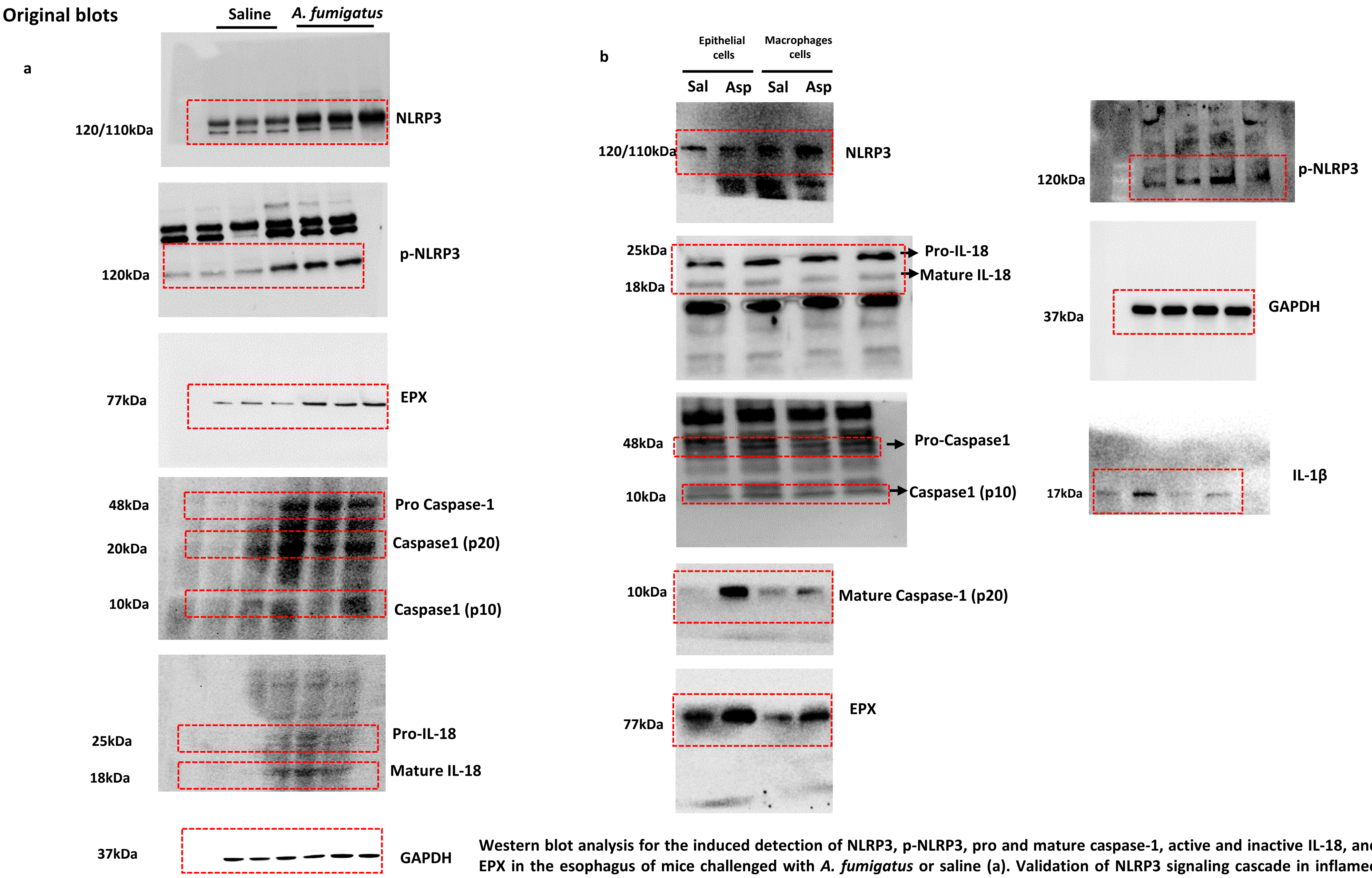

Western blot analysis for the induced detection of NLRP3, p-NLRP3, pro and mature caspase-1, active and inactive IL-18, and EPX in the esophagus of mice challenged with *A. fumigatus* or saline (a). Validation of NLRP3 signaling cascade in inflamed esophagus isolated epithelial cells and macrophages are analyzed and shown (b).

Supplementary Figure 11- Original blots

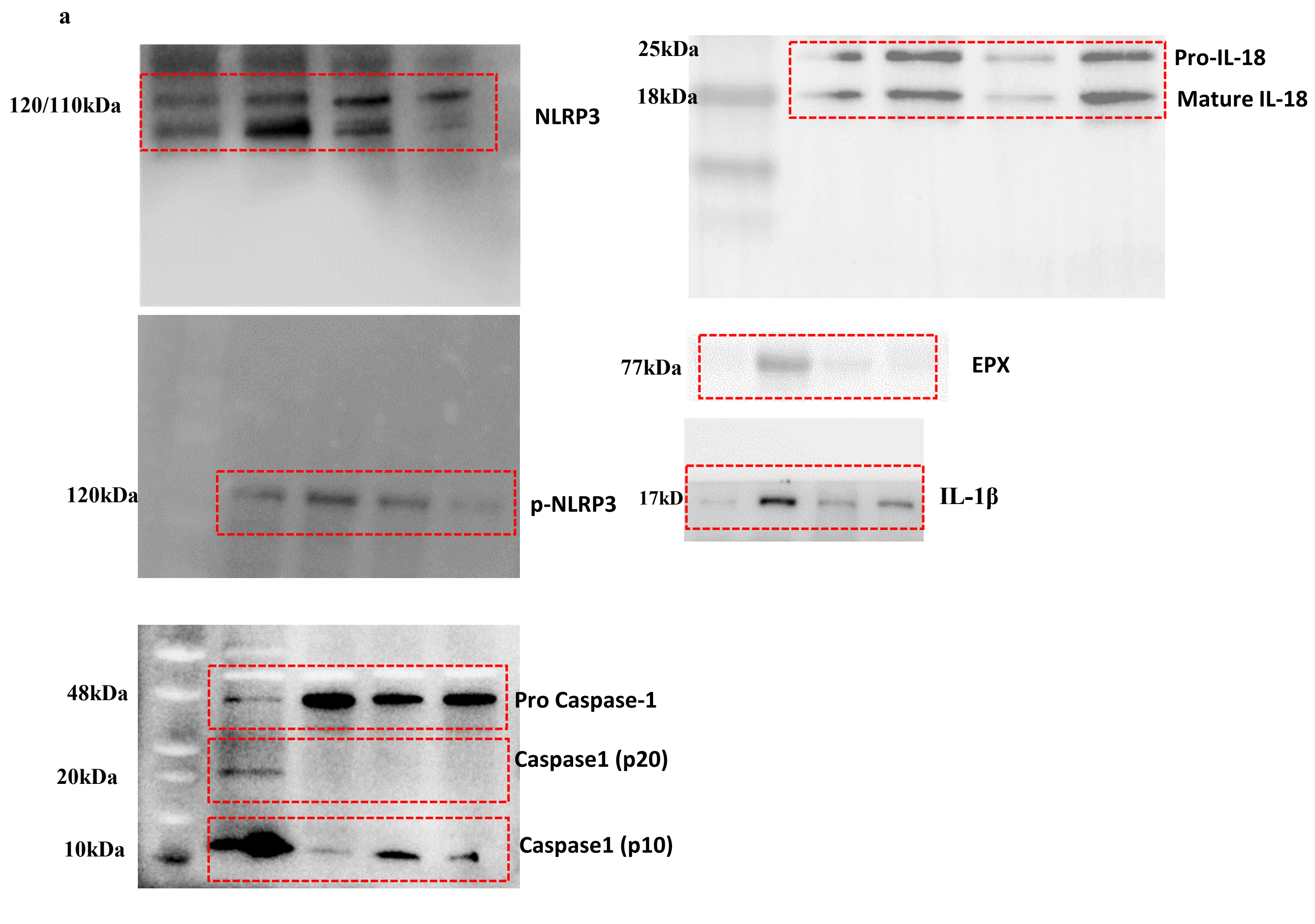

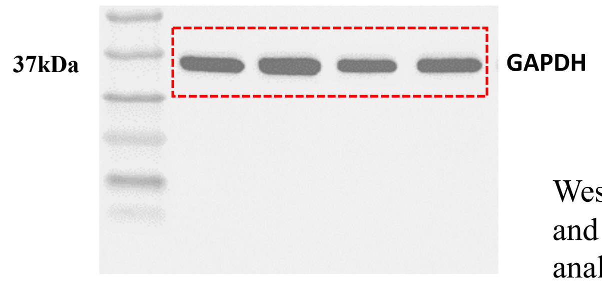

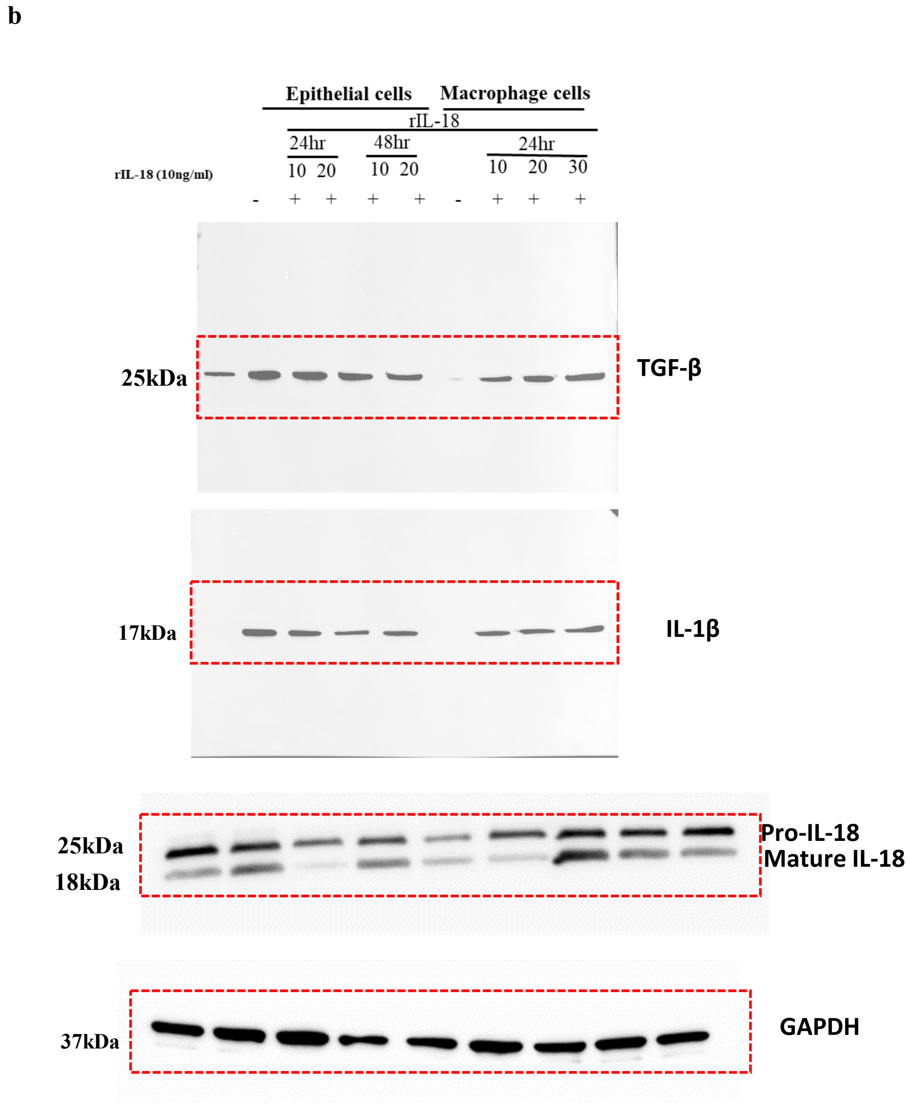

Western blot and densitometry analysis to confirm that epithelial cells and macrophages are the source of NLRP-3 regulated IL-18, Caspase, IL-18 and EPX from saline and Corn sensitized/ corn challenged mice inflamed esophagus isolated epithelial cells and macrophages (a). Western blot analysis on rIL-18 exposed isolated esophageal epithelial cell and macrophages from naïve mice (b).

Supplementary Figure 12- Original blots

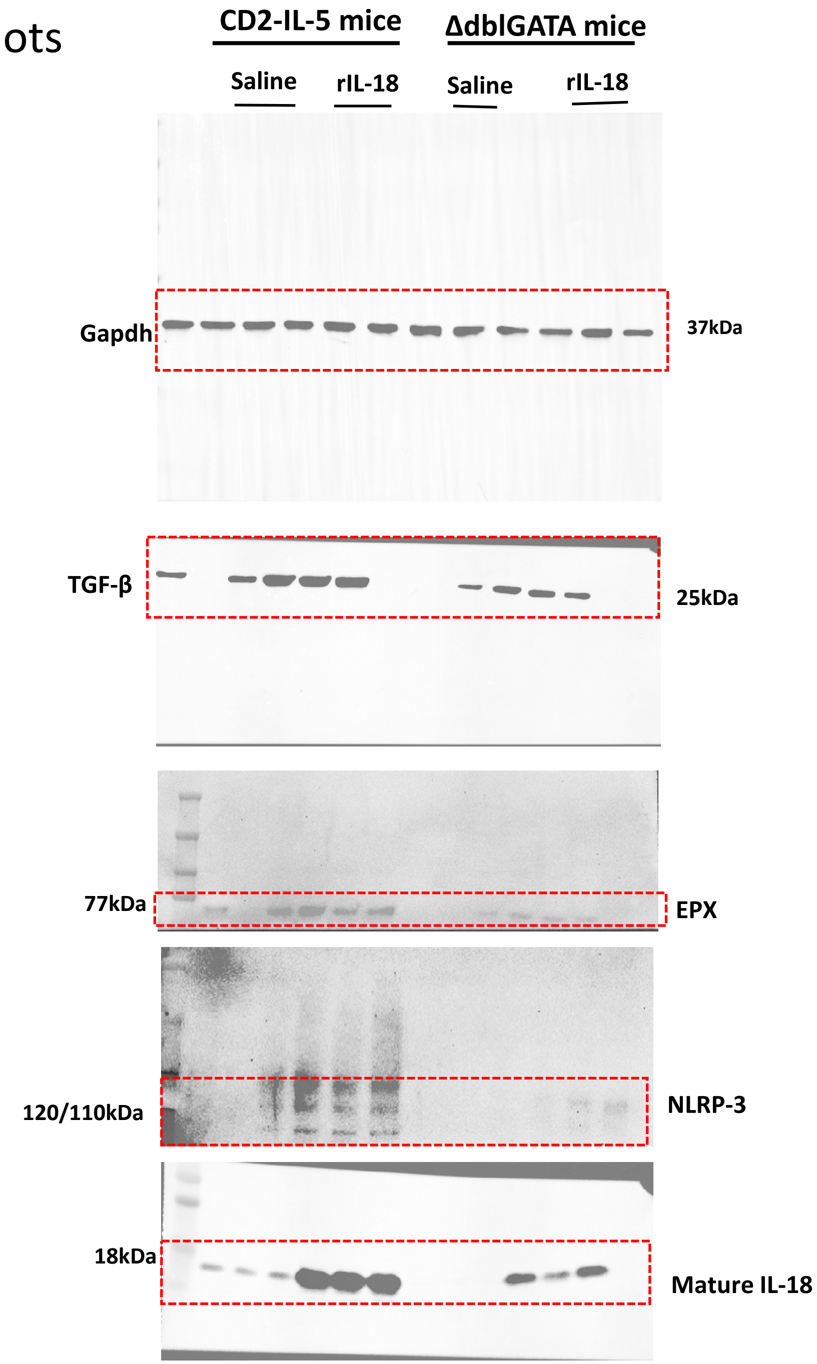

The Western blot analysis of rIL-18 treated mice esophageal extract in CD2-IL-5 mice and ΔdblGATA mice.

Suppl. Figure 13- Original blots

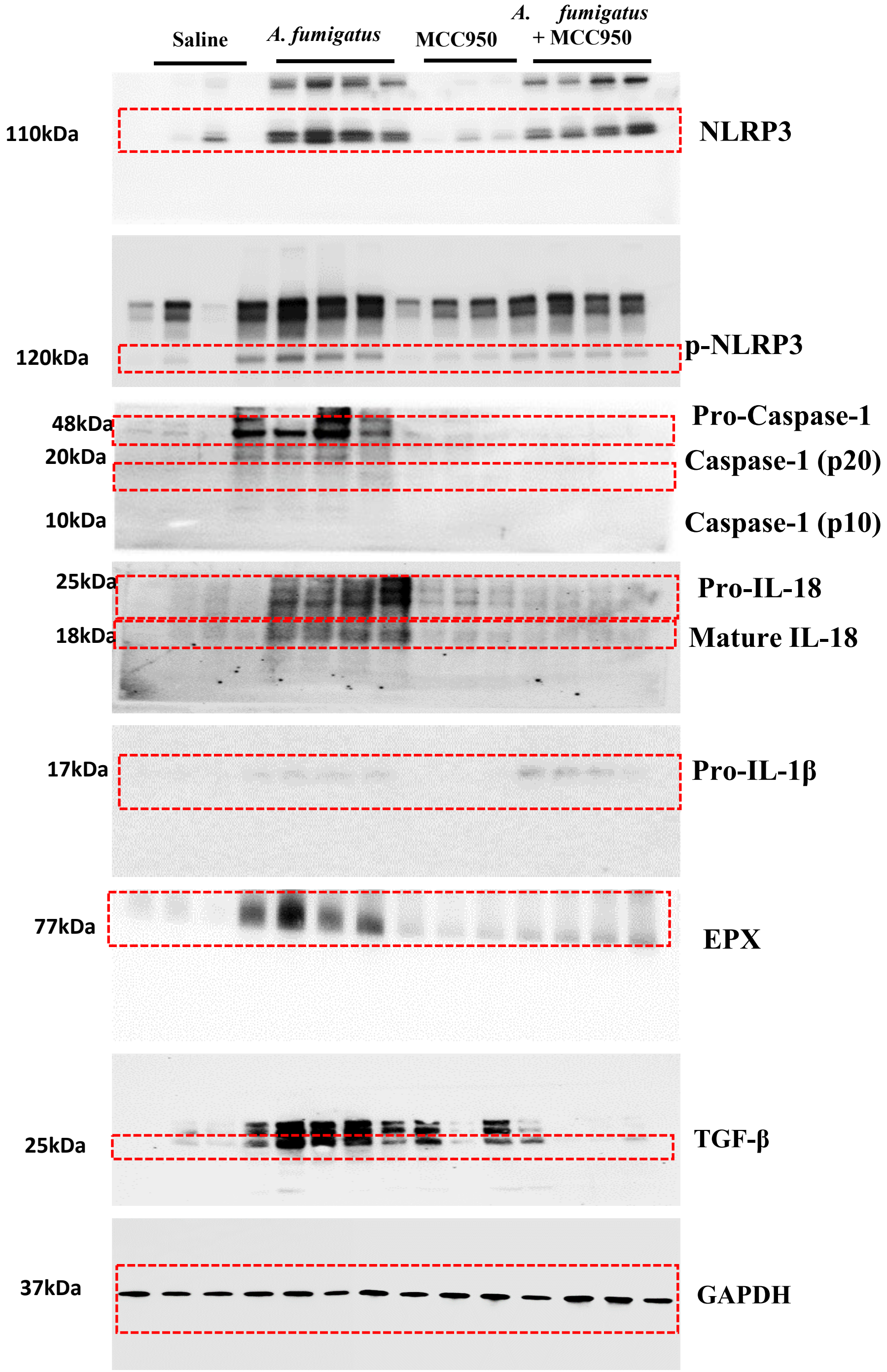

Western blot analysis of NLRP3, p-NLRP3, IL-18-caspase-1, IL-18, IL-1β, EPX and TGF-β in *A. fumigatus* challenged mice and MCC950 + *A. fumigatus* treated mice

Patients	Age	Gender	Esophageal disease	Allergic diseases	Current treatment
1	9	M	NL	Asthma	None
2	11	F	NL	Unknown	None
3	7	F	NL	None	H1R
4	4	M	NL	Rhinitis	LTRA
5	11	M	NL	None	PPI
6	9	M	NL	Asthma	None
7	12	M	NL	None	None
8	13	F	NL	Unknown	PPI
9	10	M	NL	Asthma	INHGC
10	12	M	NL	None	None
11	8	F	NL	Rhinitis	None
12	14	F	EoE	Asthma	None
13	10	M	EoE	None	None
14	11	F	EoE	Food Allergy	None
15	10	F	EoE	Asthma/Eczema	None
16	11	M	EoE	None	None
17			EoE	Asthma/Rhinitis	None
18	5	M	EoE	None	INHGC
19	6	M	EoE	None	PPI
20	7	M	EoE	Rhinitis/Asthma	B2ARA
21	8	M	EoE	None	Elementary diet therapy, PPI
22	11	M	EoE	Unknown	PPI
23	20	M	EoE	Rhinitis	Elementary diet therapy, PPI
24	20	F	EoE	Asthma	PPI
25	7	M	EoE	Asthma/Eczema	INHGC
26	8	M	EoE	Food allergy	Elementary diet therapy

Supplementary Table 1. Patients clinical and pathological characteristics

Abbreviations: NL = normal, M = male, F = female, EoE = eosinophilic esophagitis; LTRA, leukotriene receptor antagonist; H1RA, H1-receptor antagonist; INHGC, inhaled glucocorticoid; B2ARA, 2 adrenergic receptor antagonists; PPI, proton pump inhibitor.

Supplementary Figures.

Supplementary Fig. 1 Detailed photomicrographs of immunofluorescence analysis of NLRP3/IL-18 and esophageal fibrosis in EoE.

Schematic presentation of *A. fumigatus*-induced experimental EoE mouse model (a). A representative photomicrograph of individual immunofluorescence analyses of NLRP3, IL-18, F4/80, and NLRP3 expression with merged photomicrograph illustrating their expression in macrophages and epithelial cells of mice challenged with saline vs *A. fumigatus* (b-e). Induced detection of collagen (f, i-ii) and TGF-β^+ cells (g, i-ii) in saline- and *A. fumigatus*-challenged mice with morphometric analysis (f iii, g iii). Data are expressed as mean ± SD, *p < .05; **p < .001; ***p <.001. Photomicrographs are presented at 400x original magnification (scale bar 20 µm).

Supplementary Fig. 2 Detailed photomicrographs of immunofluorescence analysis of NLRP3/IL-18 and esophageal fibrosis in EoE.

Schematic protocol presentation of food (corn) allergen-induced EoE mouse model (a). A representative photomicrograph of individual immunofluorescence analyses of NLRP3, IL-18, F4/80, and NLRP3 expression with merged photomicrograph illustrating their expression in macrophages and epithelial cells of saline- and *Corn*-challenged mice (b-e). Western blot analysis of naïve mice isolated epithelial cells and macrophages exposed with rIL-18 to confirm whether epithelial cells and macrophages induce NLRP3 and Caspase1 and profibrotic cytokines (f). Photomicrographs are presented at 400x original magnification (scale bar 20 µm)

.

Supplementary Fig. 3 Transcript expression of *NLRP3*, *IL-13*, *IL-4*, *IL-5*, *IL-18*, and *IL-1β* following *A. fumigatus* challenge in wild type and *IL-18*$^{-/-}$ mice.

The relative mRNA normalized with GAPDH levels of *NLRP3*, *IL-13*, *IL-4*, *IL-5*, *F4/80*, *IL-1β*, and *IL-18* in *A. fumigatus*-challenged wild-type and *IL-18*$^{-/-}$ mice. Data are expressed as mean ± SD, *p < .05; **p < .001; ***p <.001.

Supplementary Fig. 4 Detailed immunoassayed representative photomicrographs of anti–IL-18 neutralized mice

following *A. fumigatus*-induced experimental EoE.

A representative photomicrograph of anti-MBP, Ki-67$^+$, and TGF-β$^+$ immunostained esophageal sections from *A. fumigatus*-challenged wild-type mice following anti-IL-18 and respective matched IgG isotype control-treated mice (a-c i–iv). Masson's trichrome analysis for collagen accumulation in the esophagus of *A. fumigatus*-challenged IL-18–neutralized or IgG treated non-neutralized mice (d i–iv). Photomicrographs are shown at 400× original magnification (scale bar 20 μm). EP, epithelium; LP, lamina propria; MS, muscular mucosa; LU, lumen.

Supplementary Fig. 5 Detailed immunoassayed immunofluorescence photomicrographs and transcript analysis data in *A. fumigatus*-challenged wild-type mice and *GM-CSF*$^{-/-}$ mice.

A representative photomicrograph of individual immunofluorescence analysis of NLRP3, IL-18, F4/80, and NLRP3 expression with merged photomicrograph to show their expression in macrophages and epithelial cells of saline- and *A. fumigatus*-challenged wild-type and *GM-CSF*$^{-/-}$ mice. NLRP3-F4/80 and NLRP3-IL-18 combination in merged photographs (a-h). Photomicrographs are presented at 400x original magnification (scale bar 20 μm).

Supplementary Fig. 6 Detailed immunoassay photomicrograph of human EoE and control biopsies analyzed for NLRP3, CD-163, IL-18, cytokeratin, and caspase-1.

A representative photomicrograph of immunofluorescence analysis performed for NLPR3/CD163 (a–b i–iv); NLRP3/IL-18 (c–d i–iv); NLRP3/caspase-1 (e-f i-iv) in human control and EoE biopsies. Individual photomicrographs of NLRP3, caspase-1, cytokeratin, CD-163 and IL-18 illustrate their expression in accumulated macrophages and epithelial cells. Photomicrographs are presented at 100x of original magnification (scale bar 5 μm). A representative photomicrograph of detailed individual anti-cytokeratin and NLRP3 immunostained human biopsies tissue sections of normal and EoE patients (g-h i-iv). Photomicrographs are presented at 400x of original magnification (scale bar 20 μm).

Supplementary Fig. 7 Analysis of anti-TGF-β and anti-Ki-67 immunostaining in mice following treatment with *Aspergillus*, *Aspergillus* with MCC950, *Aspergillus* with BHB, *Aspergillus* with VX-765, corn alone, and corn with MCC950 with morphometric analysis.

Schematic protocol presentation of MCC950 or BHB or VX-765 treated, and food (corn) allergen induced EoE mouse model (a i-ii). Detailed photomicrograph of Ki-67, TGF-β $^{+}$ cells are increased tissue sections of *A. fumigatus*-challenged mice (b, i) and reduced in mice treated with *A. fumigatus* + NLRP3 inhibitors (MCC950 and BHB; b ii–iii). Reduced Ki-67^{+} expression in esophageal epithelial cells in mice treated with NLRP3 inhibitors (MCC950, BHB, VX-765) after challenge with *A. fumigatus*/corn compared to untreated challenged mice (b, d, f, h). Morphometric quantification analysis of TGF-β and Ki-67 expressed as cells/mm^2 (c, e, g, i). Photomicrographs are presented at 400x original magnification (scale bar 20 μm).

Supplementary Fig. 8 Detailed photomicrographs of immunofluorescence analysis following treatment with NLRP3 inhibitors (MCC950, BHC) in *Aspergillus*-induced EoE mouse models.

A representative image of individual NLRP3 and IL-18, immunoassayed and double positive cells of NLRP3/IL18 in mice challenged with *A. fumigatus* following NLRP3 inhibitors (MCC950, BHB (i-ii a-i). A representative image of individual NLRP3-F4/80 immunoassayed and double positive cells of NLRP3/IL18 in mice challenged with saline or corn extract and treated with the NLRP3 inhibitor MCC950 (iii j-l). Photomicrographs are presented at 400x original magnification (scale bar 20 μm).

Supplementary Fig. 9. Detailed photomicrographs of immunofluorescence analysis following caspase-1 (VX765) inhibitor treatment in *Aspergillus*-induced EoE.

A representative image of individual NLRP3-F4/80 immunoassayed and double positive cells of NLRP3/IL18 in mice challenged with saline or corn-extract and treated with the caspase inhibitor VX765 (a–d). Photomicrographs are presented at 400x original magnification (scale bar 20 μm).
